# The prevalence and predictors of clinical breast cancer screening in Sub-Saharan African countries: a multilevel analysis of Demographic Health Survey

**DOI:** 10.3389/fpubh.2024.1409054

**Published:** 2024-09-13

**Authors:** Aklilu Habte Hailegebireal, Habtamu Mellie Bizuayehu, Biruk Bogale Wolde, Lire Lemma Tirore, Beshada Zerfu Woldegeorgis, Gizachew Ambaw Kassie, Yordanos Sisay Asgedom

**Affiliations:** ^1^School of Public Health, College of Medicine and Health Sciences, Wachemo University, Hosanna, Ethiopia; ^2^Faculty of Health and Environmental Sciences, Auckland University of Technology, Auckland, New Zealand; ^3^Faculty of Medicine, School of Public Health, The University of Queensland, Brisbane, QLD, Australia; ^4^Department of Public Health, College of Medicine and Health Sciences, Mizan Tepi University, Mizan, Ethiopia; ^5^Department of Health Informatics, School of Public Health, College of Medicine and Health Sciences, Wachemo University, Hosanna, Ethiopia; ^6^Department of Internal Medicine, College of Health Sciences and Medicine, Wolaita Sodo University, Wolaita Sodo, Ethiopia; ^7^Department of Epidemiology and Biostatistics, College of Health Sciences and Medicine, Wolaita Sodo University, Wolaita Sodo, Ethiopia; ^8^Department of Epidemiology, College of Health Sciences and Medicine, Wolaita Sodo University, Wolaita Sodo, Ethiopia

**Keywords:** breast cancer, screening, Sub-Saharan Africa, multilevel, determinants

## Abstract

**Background:**

Despite a higher rate of breast cancer in sub-Saharan Africa (SSA), efforts to treat the disease through breast cancer screening are suboptimal, resulting in late diagnosis of breast cancer and poor outcomes. Several studies have been conducted in SSA countries about screening uptake, yet they addressed country or sub-country level data and did not consider both individual and beyond-individual factors related to screening. Hence, pooled prevalence as well as multilevel correlates of screening in the region is sparse, which have been addressed by this study using the most recent data among women with SSA.

**Methods:**

This study was conducted using the Demographic Health Survey data (2013–2022) from six countries, and a total weighted sample of 95,248 women was examined. STATA version 16 was used for the data analysis. Multilevel mixed-effects logistic regression was performed and significant predictors were reported using adjusted odds ratios (aOR) with 95% confidence intervals (95% CI).

**Results:**

The overall weighted prevalence of clinical breast cancer screening was 14.23% (95% CI: 13.97–14.75), with Namibia and Tanzania having the highest (24.5%) and lowest (5.19%) screening rates, respectively. Higher breast cancer screening uptake was observed among women of advanced age (35–49) [aOR = 1.78; 95% CI: 1.60, 1.98], had higher educational levels [aOR = 1.84; 95% CI: 1.66, 2.03], cohabited [aOR = 1.37; 95% CI: 1.21, 1.55], in the richest wealth quintile [aOR = 2.27; 95% CI: 1.95, 2.64], urban residents [aOR = 1.21; 95%CI: 1.10, 1.33], multiparous [aOR = 1.47; 95% CI: 1.30, 1.68], visited health facilities [aOR = 1.64; 95% CI: 1.52, 1.76], and read newspapers [aOR = 1.78; 95%CI: 1.60, 2.15].

**Conclusion:**

The prevalence of clinical breast cancer screening was low (14%). Strengthening awareness campaigns, improving healthcare infrastructure, health education, universal health coverage, and screening program access, with a focus on rural areas, women who lack formal education, and low socioeconomic status, are critical to increasing breast cancer screening rates and equity. Scale-up local and regional collaborations and the involvement of media agencies in the implementation of screening programs, advocacy, dissemination of information, and integration of screening programs with their routine care, such as perinatal care, can boost the screening. The existing health service delivery points also need to focus on integrating breast cancer screening services with routine care such as perinatal care.

## Background

Breast cancer occurs when breast cells proliferate uncontrollably and form tumors (1, 2). Breast cancer among women was the first leading cancer worldwide in 2020, with 2.3 million cases diagnosed and 685,000 deaths. It will also continue to be the leading cancer over the next two Decades, estimated to increase by about 50% in 2040 (over 3 million cases and 1 million deaths) (3). Breast cancer affects one in every 20 women worldwide, and as many as one in every eight in high-income nations (4). It is the most prevalent cancer in Africa, with an estimated 497,127 cases and 309,637 deaths in 2020, and the burden of breast cancer is projected to about double by 2040 (946,424 cases and 598,511 deaths) (3). Sub-Saharan Africa (SSA) has the greatest mortality-to-incidence ratio, with around 80% of cases presenting with locally advanced and metastatic disease upon diagnosis and poor survival (3, 5).

Breast cancer can be prevented through effective intervention of modifiable risk factors and early identification of the disease by screening (4, 6). The WHO launched the Global Breast Cancer Initiative (GBCI) in 2021, intending to reduce breast cancer incidence by 2.5% per year by 2040 through health promotion, early detection by screening, and treatment (comprehensive breast cancer management) (7). Breast cancer screening offers tests to asymptomatic women to seek medical attention (8). Screening could be performed in either an opportunistic or systematic approach. Opportunistic screening takes place when a woman without signs of breast cancer is referred for screening tests outside of a formal program, which aids in the early detection of non-palpable breast malignancies (9). Systematic screening, on the other hand, refers to a formal screening process for a specified population implemented by a health facility, or regional or national government (Ministry of Health) (8, 10). This strategy is most likely to accomplish early diagnosis in a large portion of the population, but it is also the most costly screening approach (10).

Screening can be performed using clinical breast examination (CBE), breast self-examination, or mammography (8). A Clinical Breast cancer screening is performed through a detailed history, physical examination (inspection and palpation of the breast in various positions), and lymph node examination by the clinician or healthcare practitioner (10). It aids in assessing masses, lesions, and skin changes, distinguishing between benign and malignant lesions, determining the stage of disease, counseling, planning effective therapy, and documenting clinical data for audit and follow-up (10–12).

Although breast cancer has become a public health problem in SSA, reducing the disease through screening is inadequate, resulting in late diagnosis of breast cancer and poor outcomes (13–15). A multitude of factors at the individual, system, or community level could contribute to low access to and use of breast cancer screening. Lack of awareness about breast cancer or the screening process, fear of the screening process or being diagnosed with cancer, financial concerns and screening associated costs, stigma and poor attitude about the screening, misconceptions, low health education material access, low training opportunities to healthcare providers, inequity in resource allocation, distance to the screening program, low health system commitment, and poor integration of the program into the existing health system contribute to poor screening uptake in the region (16–19).

Although several studies have been conducted in SSA countries to address breast cancer screening, they were undertaken at one country or sub-country level and did not consider both individual and beyond individual factors related to screening. Evidence about the pooled prevalence as well as multilevel correlates of screening in the region is sparse (20–22). Hence, the current study addressed the evidence gap by estimating the prevalence and multilevel factors related to screening among women in SSA using the most recent standard Demographic Health Survey data (2013–2022). The knowledge will assist public health planners and policymakers in devising targeted intervention strategies to improve screening rates by working on individual and community level determinants of screening. In addition, studying prevalence using the most recent DHS data enables the evaluation of the influence of measures such as awareness campaigns, screening programs, and policy changes aimed at raising screening uptake.

## Methods

### Data source, population, and study period

The study was based on the appended woman (IR) file of the most recent Demographic and Health Surveys (DHS) of six SSA countries (Burkina Faso, Cote d’Ivoire, Kenya, Lesotho, Namibia, and Tanzania). The study comprised all women who had complete information on the outcome of interest (clinical breast cancer screening) a total of 95,248 women (Table 1).

**Table 1:** Description of the countries included in the analysis with their respective sample size, 2013–2022.

Country	Year	Weighted sample size (%)
Burkina Faso	2021	17,638 (18.5)
Cote d’Ivoire	2021	14,866(15.6)
Kenya	2022	16,649 (17.5)
Lesotho	2014	6,585 (6.9)
Namibia	2013	9,131 (9.6)
Tanzania	2022	30,379 (31.9)
Total	2013–2022	95,248 (100.0)

The study participants were selected using a two-stage stratified cluster sampling procedure, and data were collected through face-to-face interviews. The DHS Sampling and Household Listing Manual provides a full description of the sampling technique (23).

### Measurement of variables

#### Outcome variable

The type of breast screening measured in DHS and also implemented in the included countries was a clinical breast examination, which was assured if women answered yes to any of the following questions: “Have you ever had a breast cancer screening?” “Has a doctor or other health professional examined your breast to detect or check for breast cancer?” The response from any of the questions was dichotomised as yes = 1 or no = 0 (24).

### Explanatory variables

Potential variables at the individual and community levels were identified by considering prior literature on the area of interest (20–22, 24). Individual-level factors were: Women’s age (15–19, 20–34, and 35–49), educational status (no education, priMary, secondary, and higher education), Marital status (cohabited, unmarried, and non-marital relation), family size (≤5 and > 5), wealth index (poorest, poorer, middle, richer, and richest), parity (nulliparous, primiparous, multiparous, and grand multiparous), contraceptive uptake (user or non-user), recent sexual activity (never had sex, not active in the last 4 weeks, and active in the last 4 weeks), ease of seeking medical care due to distance, money (big problem or not a big problem), media (radio, TV, Newspaper) exposure (not at all, less than once a week, and at least once a week), autonomy in Decision-making (low, medium, higher) (25, 26), went to health facility within a year (yes, or no) and enrolment in health insurance schemes (yes, or no). Community-level factors were shared by all women living in the same community (cluster), such as residence (urban or rural) and country.

### Statistical analysis and data management

STATA version 16 was used for the data analysis. Weighting was performed before any statistical analysis to ensure survey representativeness and reliable statistical estimations. Frequencies and percentages were computed to determine the characteristics of the respondents. Given that the DHS data were hierarchical, we used multilevel modeling. First, a multilevel bivariable logistic regression was performed to examine the association between each explanatory variable and the outcome variable. Variables with *p* < 0.25 were added into multilevel mixed-effect logistic regression. A multilevel multivariable logistic regression analysis was used to identify significant predictors of CBE. Statistical significance was declared at *p* < 0.05. There was no multicollinearity among the variables (the VIF ranged from 1.04 to 1.78, with a mean of 1.19).

### Model building and selection

Four models were constructed for multilevel binary logistic regression analysis. The first model was a null model without explanatory variables to determine the extent of cluster variation in breast cancer. The second and third models were adjusted for individual and community-level factors independently. The fourth (full) model was fitted for both individual and community-level variables simultaneously. The intraclass correlation coefficient (ICC) and proportionate change in variance (PCV) were estimated to quantify the random effects in each model (variability in CBE between and across clusters).

$ICC=\frac{var\left(b\right)}{Var\left(b\right)+Var\left(w\right)}$ , where Var (b) is the variance at the group level and Var(w) is the predicted individual variance component, which is π^2^/3 ≈ 3.29.

Proportional Change in Variance (PCV) was estimated as

$PCV=\frac{\left(Va-Vb\right)}{Va}*100$ , where V_a_ is the variance of the initial model (null model) and V_b_ = variance of the subsequent models (models 2, 3, and 4).

Model comparisons were made based on deviance [−2Log-Likelihood Ratio (LLR)] because the models were nested models, and the model with the lowest deviance was the best-fitted model for the data.

## Results

### Background characteristics of the respondents

This study analysed a total weighted sample of 95,248 women with a mean (±SD) age of 29.26 (±9.96), with the majority (39.0%) belonging to the age group 15–24 years (Table 2). Tanzania and Lesotho had the largest and smallest sample sizes, with 31.9 and 6.9%, respectively. More than half (58.0%) of women lived in rural areas, and 41.5% attained secondary or higher education. The majority of women (38.9%) were multiparous (with 2–4 living children), and more than two-thirds (64.7%) did not use contraception. Regarding media exposure, 76.9, 46.2, and 38.5% of women had never read a newspaper, watched television, or listened to the radio, respectively. Only 55.4% of women visited health facilities within the last 12 months (Table 2).

**Table 2:** Distribution of background characteristics of study participants: clinical breast cancer screening practice and bivariable analysis, SSA, 2013–2022.

Variable categories	Total (*N* = 95,248)	Received clinical breast cancer screening [Frequency (%)]	cOR(95% CI)	*p*-value
Countries				
Burkina Faso	17,638(18.5)	4,320(24.5)	6.38(5.56, 7.33)	<0.001
Cote d’Ivoire	14,866(15.6)	2,597(17.5)	3.70(3.17, 4.32)	<0.001
Kenya	16,649(17.5)	2,315(14.0)	2.68(2.34, 3.07)	<0.001
Lesotho	6,585(6.9)	641(9.7)	2.04(1.75, 2.39)	<0.001
Namibia	9,131(9.6)	2,105(23.0)	5.66(4.86, 6.60)	<0.001
Tanzania	30,379(31.9)	1,578(5.2)	Ref.	
Current age				
15–24	37,127(39.0)	3,352(9.0)	Ref.	
25–34	29,069(30.5)	4,845(16.7)	2.07(1.92, 2.23)	<0.001
35–49	29,052(30.5)	5,358(18.4)	2.37(2.19, 2.56)	<0.001
Marital status				
Cohabited	56,431(59.2)	9,317(16.5)	1.95(1.79, 2.13)	<0.001
Not in union^*^	8,743(9.2)	1,235(14.1)	1.59(1.40, 1.80)	<0.001
Unmarried	30,074(31.6)	3,003(10.0)	Ref.	
Educational status				
No education	23,868(25.0)	3,505(14.7)	Ref.	
Primary	31,842(33.4)	3,014(9.8)	0.67(0.54, 0.95)	0.013
Secondary and higher	39,538(41.5)	7,036(17.8)	1.19(1.08, 1.30)	0.021
Residence				
Urban	40,026(42.0)	7,619(19.0)	2.03(1.83, 2.24)	<0.001
Rural	55,222(58.0)	5,936(10.8)	Ref.	
Family size				
≤5 member	47,804(50.2)	7,018(14.7)	1.05(0.98, 1.12)	0.112
>5 member	47,444(49.8)	6,537(13.8)	Ref.	
Wealth index combined				
Poorest	15,447(16.2)	1,159(7.5)	Ref.	
Poorer	16,545(17.4)	1,741(10.5)	1.45(1.29, 1.63)	<0.001
Middle	18,106(19.0)	2,292(12.7)	1.81(1.59, 2.06)	<0.001
Richer	20,993(22.0)	3,234(15.4)	2.34(2.04, 2.68)	<0.001
Richest	24,156(25.4)	5,127(21.2)	3.51(3.05, 4.05)	<0.001
Sex of household head				
Male	67,033(70.4)	9,529(14.2)	Ref.	
Female	28,215(29.6)	4,026(14.3)	0.99(0.92, 1.06)	0.778
Parity				
Nulliparous	27,136(28.5)	2,267(8.4)	Ref.	
Primiparous	16,290(17.1)	2,685(16.5)	2.27(2.05, 2.52)	<0.001
Multiparous	37,011(38.9)	6,549(17.7)	2.56(2.32, 2.81)	<0.001
Grand multiparous	14,811(15.5)	2,054(13.9)	2.01(1.80, 2.25)	<0.001
Contraceptive utilization				
Non-user	61,606(64.7)	7,438(12.1)	Ref.	
Users	33,642(35.3)	6,117(18.2)	1.62(1.53, 1.72)	<0.001
Recent sexual activity				
Never had sex	13,449(14.1)	568(4.2)	Ref.	
Not active in the last 4 weeks	48,937(51.4)	7,652(15.6)	4.47(3.81, 5.25)	<0.001
Active in the last 4 weeks	32,862(34.5)	5,335(16.2)	4.62(3.94, 5.40)	<0.001
Visit health facility within the last 12 months				
Yes	52,796(55.4)	9,565(18.1)	2.18(2.03, 2.34)	<0.001
No	42,452(44.6)	3,989(9.4)	Ref.	
Reading newspaper				
Not at all	73,275(76.9)	9,355(12.8)	Ref.	
Less than once a week	12,713(13.4)	1,901(15.0)	1.20(1.10, 1.31)	0.016
At least once a week	9,260(9.7)	2,299(24.8)	2.16(1.95, 2.39)	<0.001
Listening to a radio				
Not at all	36,701(38.5)	4,281(11.7)	Ref.	
Less than once a week	20,204(21.2)	2,842(14.1)	1.23(1.12, 1.35)	<0.001
At least once a week	38,343(40.3)	6,431(16.8)	1.53(1.42, 1.65)	<0.001
Watching television				
Not at all	44,014(46.2)	4,671(10.6)	Ref.	
Less than once a week	13,664(14.4)	1,866(13.7)	1.34(1.22, 1.46)	<0.001
At least once a week	37,569(39.4)	7,018(18.7)	1.85(1.71, 2.00)	<0.001
Autonomy in decision-making				
Low	21,487(22.6)	3,440(16.0)	Ref.	
Middle	8,966(9.4)	1,671(18.6)	1.12(1.00, 1.25)	0.034
High	64,794(68.0)	8,444(13.0)	0.82(0.66, 1.18)	0.231
Attitude toward wife beating				
Low	75,225(79.0)	11,349(15.1)	1.68(1.45, 1.95)	<0.001
Middle	14,083(12.2)	1,660(11.8)	1.29(1.11, 1.51)	<0.001
High	5,939(6.2)	547(9.2)	Ref.	
Distance to a health facility				
Big problem	29,840(31.3)	3,791(12.7)	Ref.	
Not a big problem	65,408(68.7)	9,764(14.9)	1.18(1.09, 1.29)	0.013
Permission to get health service				
Big problem	12,128(12.7)	1,655(13.7)	Ref	
Not a big problem	83,120(87.3)	11,900(14.3)	1.06(0.95, 1.17)	0.251
Getting money needed for treatment				
Big problem	43,542(45.7)	5,992(13.8)	Ref	
Not a big problem	51,705(54.3)	7,562(14.6)	1.06(0.99, 1.13)	0.051

^*^Divorced, widowed, and separated, cOR, Crude odds ratio.

### The overall prevalence of clinical breast cancer screening

**Figure 1:**
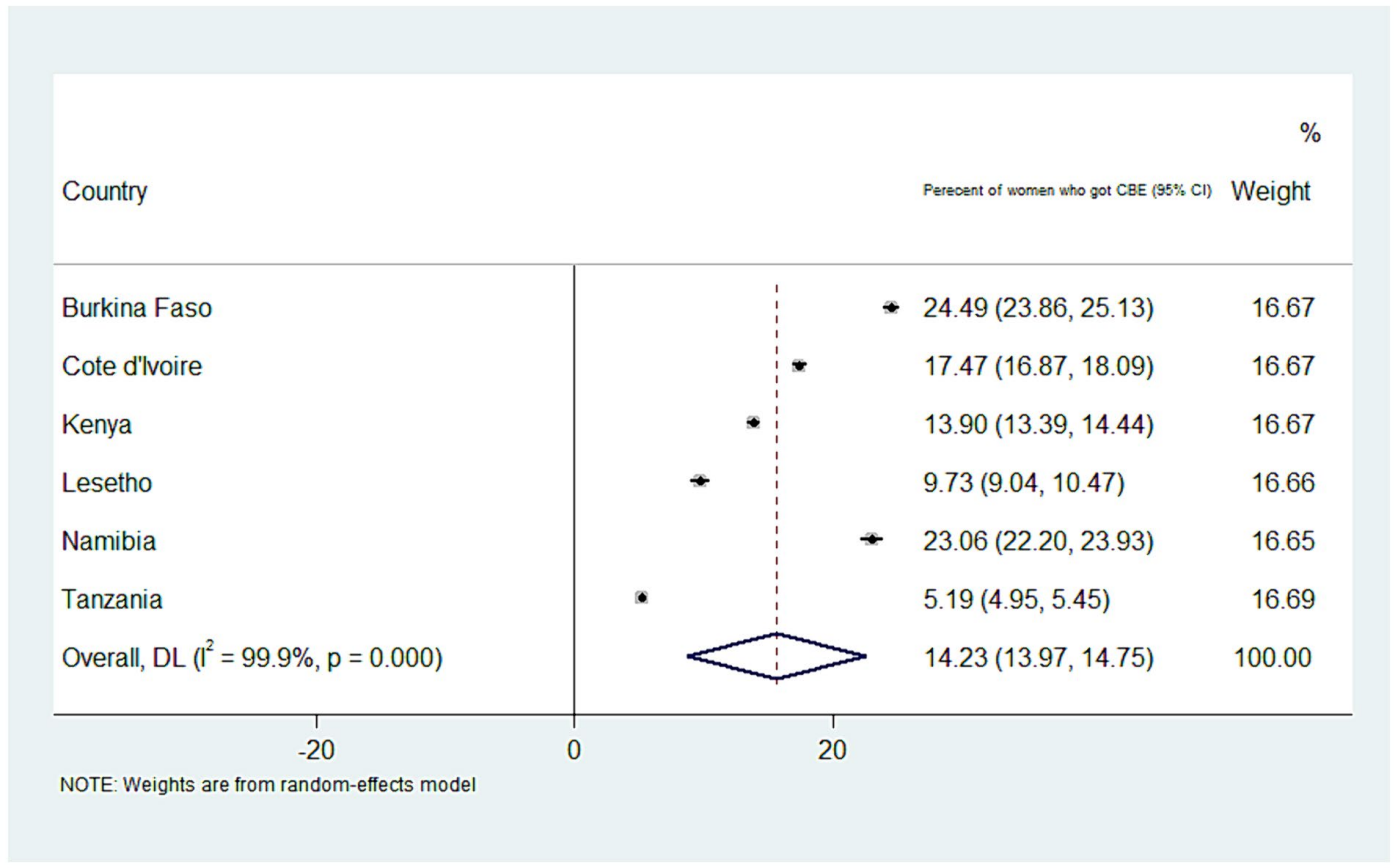
A forest plot depicting the weighted prevalence of clinical breast cancer screening in SSA countries.

The overall weighted prevalence of CBE in SSA countries was 14.23 (95% CI: 13.97, 14.75). The highest and lowest screening rates were detected in Burkina Faso and Tanzania, at 24.5% (95% CI: 23.86, 25.13) and 5.19% (95% CI: 4.95, 5.44), respectively (Figure 1).

### Random effect (measures of variation)

The null model (Model I) results revealed that the variability between clusters accounted for 15.64% of the total variation in clinical breast cancer screening across countries. In addition, individual and community-level factors separately explained 12.73 and 13.87% of the disparities in clinical breast cancer screening uptake, respectively. On the other hand, individual- and community-level factors together accounted for 37.70% of the variation observed in the null model (PCV = 37.70%) (Table 3).

**Table 3:** Random intercept variances and model fit statistics comparison of multilevel mixed effect logistic regression model.

Measures	Model I (null model)	Model II (individual-level factors)	Model III (community-level factors)	Model-IV (full model)
Random effects				
Variance	0.61	0.48	0.53	0.38
ICC	15.64%	12.73%	13.87%	10.35%
AIC	74596.0	68383.7	69174.6	64001.44
BIC	74614.9	68696.3	69250.4	64370.9
PCV	Ref.	21.31%	13.11%	37.70%
Model fitness				
Log-likelihood	−37296.0	−34158.8	−34579.3	−31961.7
Deviance	74,592	68317.6	69158.6	63923.4

### Predictors of clinical breast cancer screening practice

Women’s age, marital status, educational level, wealth index, residence, parity, visiting health facilities, and reading newspapers were significantly associated with clinical breast cancer screening (Table 4). Women aged 35–49 were 1.78 [aOR = 1.78; 95% CI: 1.60, 1.98] times more likely to receive clinical breast cancer screening than women aged 15–24 years. The odds of screening were 1.84 [aOR = 1.84; 95% CI: 1.66, 2.03] times higher among women who attended secondary education and above than their counterparts with no formal education. The odds of having clinical breast cancer screening were 2.27 [aOR = 2.27; 95% CI: 1.95, 2.64] times higher in women who lived in the richest wealth quintile than in those who lived in the poorest. Multiparous women were 1.47 [aOR = 1.47; 95% CI: 1.30, 1.68] times more likely to be screened than nulliparous one. Similarly, the odds of being screened for breast cancer were 1.64 [aOR = 1.64; 95% CI: 1.52, 1.76] times higher among women who had visited a health facility within the last 12 months than among their non-visited counterparts. Similarly, women who read a newspaper at least once a week had a 1.64 [aOR = 1.64; 95%CI: 1.44, 1.86] greater chance of being screened than those who never listened to radio. Women living in urban areas had a 21% higher chance of receiving clinical breast cancer screening than their rural counterparts [aOR = 1.21; 95%CI: 1.10, 1.33] (Table 4).

**Table 4:** Results of a multilevel mixed-effect multivariable logistic regression analysis to identify the factors affecting the uptake of clinical breast cancer screening in SSA, 2013–2022.

Variable categories	Model II (individual-level factors)	Model III (community-level factors)	Model-IV (full model)
	aOR (95% CI)	aOR (95% CI)	aOR (95% CI)
Current age			
15–24	Ref.		Ref.
25–34	1.33(1.23, 1.45)		1.34(1.23, 1.47)
35–49	1.83(1.65, 2.04)		1.78(1.60, 1.98)
Marital status			
Cohabited	1.10(0.97, 1.24)		1.37(1.21, 1.55)
Not in union^*^	0.98(0.85, 1.13)		1.30(1.12, 1.52)
Unmarried	Ref.		Ref.
Educational status			
No education	Ref.		Ref.
Primary	0.78(0.62, 1.04)		1.21(1.10, 1.33)
Secondary and higher	1.16(1.05, 1.28)		1.84(1.66, 2.03)
Family size			
≤5 members	0.91(0.84, 1.09)		1.03(0.96, 1.10)
>5 members	Ref.		Ref.
Wealth index combined			
Poorest	Ref.		Ref.
Poorer	1.42(1.26, 1.59)		1.30(1.17, 1.44)
Middle	1.65(1.45, 1.87)		1.49(1.33, 1.67)
Richer	1.97(1.72, 2.26)		1.72(1.52, 1.94)
Richest	2.74(2.36, 3.18)		2.27(1.95, 2.64)
Parity			
Nulliparous	Ref.		Ref.
Primiparous	1.29(1.15, 1.44)		1.38(1.23, 1.55)
Multiparous	1.36(1.21, 1.54)		1.47(1.30, 1.68)
Grand multiparous	1.11(0.96, 1.29)		1.30(1.12, 1.53)
Contraceptive utilization			
Non-user	Ref.		Ref.
Users	1.20(1.13, 1.28)		1.18(0.91, 1.27)
Visit health facility within the last 1 year			
Yes	1.72(1.60, 1.84)		1.64(1.52, 1.76)
No	Ref.		Ref.
Reading newspaper			
Not at all	Ref.		Ref.
Less than once a week	1.11(1.01, 1.21)		1.19(0.98, 1.31)
At least once a week	1.72(1.53, 1.93)		1.64(1.44, 1.86)
Listening to a radio			
Not at all	Ref.		Ref.
Less than once a week	1.02(0.92, 1.12)		1.02(0.93, 1.12)
At least once a week	1.04(0.96, 1.12)		1.05(0.98, 1.14)
Watching television			
Not at all	Ref.		Ref.
Less than once a week	1.08(0.98, 1.18)		1.07(0.98, 1.18)
At least once a week	1.16(1.07, 1.26)		1.03(0.95, 1.12)
Autonomy in decision-making			
Low	Ref.		Ref.
Middle	1.25(1.08, 1.32)		1.14(1.05, 1.25)
High	1.35(1.10, 1.46)		1.24(1.11, 1.39)
Attitude toward wife beating			
Low	1.22(1.06, 1.40)		1.01(0.88, 1.16)
Middle	1.14(0.98, 1.32)		1.11(0.96, 1.29)
High	Ref.		Ref.
Distance to a health facility			
Big problem	Ref.		Ref.
Not a big problem	0.97(0.89, 1.06)		0.94(0.86, 1.02)
Permission to get health service			
Big problem	Ref.		Ref.
Not a big problem	1.00(0.89, 1.13)		1.13(1.00, 1.27)
Getting money needed for treatment			
Big problem	Ref.		Ref.
Not a big problem	0.88(0.82, 0.95)		1.08(0.98, 1.16)
Countries			
Burkina Faso		6.64(5.81, 7.59)	8.78(7.68, 9.85)
Cote d’Ivoire		3.18(2.77, 3.66)	5.04(4.40, 5.79)
Kenya		2.70(2.38, 3.07)	2.49(2.20, 2.83)
Lesotho		2.09(1.79, 2.44)	1.59(1.36, 1.85)
Namibia		5.04(4.40, 5.76)	4.31(3.73, 4.98)
Tanzania		Ref.	Ref.
Residence			
Urban		1.96(1.82, 2.12)	1.21(1.09, 1.33)
Rural		Ref.	Ref.

Key: 1: Reference category; AOR, Adjusted odds ratio; ^**^ Statistically significant at *p* < 0.05.

## Discussion

The pooled prevalence of clinical breast cancer screening in SSA was 14.23(95% CI: 13.97, 14.75), which varied significantly across countries. Higher screening rate was observed among women with advanced age (35–49 years), urban residents, higher educational levels, and richest wealth quintile, in Marital relationships (cohabited), multiparous, visiting health facilities, and reading newspapers. This prevalence is higher than a previous study conducted in developing countries (11.4%) (27) but lower than studies conducted in 14 low-resource countries (15.41%) (28), Thailand (40.1%) (29), Iran (29.3%) (30), and Malaysia (77.7%) (31). Low coverage in SSA May be due to a lack of awareness about breast cancer and the importance of early detection, limited healthcare infrastructure (shortage of healthcare facilities, trained healthcare professionals, and diagnostic equipment), financial constraints, possible stigmatization and fear associated with cancer, and low prioritization by governments due to multiple competing priorities, including infectious diseases (17, 24, 32, 33). Thus, governments and healthcare stakeholders must work together to achieve the global breast cancer initiative implementation framework, which includes assessing, enhancing, and scaling up services for early detection and screening (7). In addition, it is essential to implement comprehensive strategies: raising awareness about breast cancer, improving healthcare infrastructure, providing affordable or free screening services, covering screening related costs for transportation and opportunity cost loss while attending the screening, addressing cultural and social barriers, and increasing the number of trained healthcare professionals in breast cancer detection and management.

Women with a higher level of education are more likely to receive screening services, which is supported by previous studies (22, 24, 34–37). This could be because women with higher education levels have better health literacy with more access to information about breast cancer (risk factors and symptoms) and the necessity of regular examinations and early detection. In addition, these groups are expected to be more empowered and autonomous in decision-making; more confident in advocating for their health needs; and taking proactive actions to obtain screening services. This finding underlines the necessity of broadening access to breast cancer screening among women with no formal education. As supported by multicountry studies (24, 28, 37) and nationwide studies in India (38), Thailand (29), and Botswana (39), the likelihood of screening was higher among women living in the richest wealth quintile. This might be due to women in the richest wealth quintile have greater access to healthcare facilities, including screening and diagnostic services, as well as health information, education, and awareness programes (39).

The odds of breast cancer screening were found to increase among women at a more advanced age (35–49 years), which has been supported by studies conducted elsewhere (24, 39). This could be because as an increase in women age, their risk of developing breast cancer increases, and thus this age range is considered an important time for screening (40). Furthermore, older women are more aware of the importance of regular breast cancer screening due to greater exposure to public awareness campaigns, healthcare professionals, friends, and family, which encourages them to seek screening. Similarly, high screening practice was observed among multiparous women. This could be explained by multiparous women may have frequent contact with healthcare providers during pregnancy and postpartum periods, boosting awareness of their health and the significance of regular screenings. In tandem with a systematic review and meta-analysis (37), and studies conducted in Iran (30), the current study revealed that women in marital relationships (cohabited) had a higher likelihood of clinical breast cancer screening practice. This could be due to cohabiting women being more health-conscious and having more access to social and financial support from their partners, family, or friends, which can motivate and remind them to take care of their health, including regular breast cancer screenings. It is vital to highlight that while multiparous and 35–49-year-old, and cohabited women are more likely to have breast cancer screening, this does not imply that nulliparous, younger, and unmarried women are at a lower risk of getting breast cancer. Rather, all women, regardless of their reproductive history, age, or marital status should be offered optimal screening schedules and options to guarantee early detection and improved treatment outcomes.

Urban residents had a higher chance of being screened for breast cancer, which is in line with studies conducted elsewhere (28, 37, 41–43). This could be because women living in urban areas are more likely to have access to health facilities (44), transportation, financial support, and information via various media, which helps them realize the importance of early detection and motivates them to go for regular screenings (45, 46). Thus, concerted efforts are needed to remove these barriers for rural women by improving healthcare resources, raising awareness, assuring universal health coverage, and providing improved transportation alternatives.

Women who read newspapers were more likely to be screened, consistent with previous studies (28, 47, 48). This might be due to they often have access to a wide range of health-related information via articles, advertisements, or awareness campaigns related to breast cancer screening, which can increase their knowledge and practice of screening. In line with some studies (47, 49), women who visited health facilities within the last 12 months had a higher chance of being screened. This might be due to women receiving information, with encouragement from healthcare providers about the necessity of regular screening.

This study has strengths and drawbacks. To the best of our knowledge, this is the first study in the SSA region to assess the prevalence and multilevel determinants of breast cancer screening using a larger sample size and the most up-to-date data. Furthermore, due to the clustering effect of the DHS data, a multilevel analysis was conducted, and the results at the individual and community levels are crucial for devising contextual interventions by clinicians and relevant stakeholders to promote breast cancer screening and advocacy in SSA. However, this study had some limitations. First, because the responses were self-reported, there was a chance of social desirability and recall bias. Second, because the data were obtained from a cross-sectional survey, establishing a causal relationship between the outcome of interest and predictors may be difficult.

## Conclusion

The rate of clinical breast cancer screening was low (14%). Higher screening was observed among women of advanced age (35–49 years), urban residents, had higher educational levels, the richest wealth quintile, multiparous, visited health facilities, and read newspapers. Strengthening awareness campaigns, improving healthcare infrastructure, health education, universal health coverage, and screening program access, with a focus on rural areas, women who lack formal education, and low socioeconomic status, are critical to increasing breast cancer screening rates and equity. Scale-up local and regional collaborations and the involvement of media agencies in the implementation of screening programs, advocacy, dissemination of information, and integration of screening programs with their routine care, such as perinatal care, can boost the screening. The existing health service delivery points also need to focus on integrating breast cancer screening services with routine care such as perinatal care.

## Data Availability

The raw data supporting the conclusions of this article will be made available by the authors, without undue reservation.
